# Optimizing equine sperm quality: an alternative to single layer centrifugation for sperm isolation

**DOI:** 10.1530/RAF-23-0081

**Published:** 2024-11-11

**Authors:** Ashlee Jade Medica, Zamira Gibb, Robert John Aitken

**Affiliations:** 1 HMRI Infertility and Reproduction Research Program, Discipline of Biological Sciences, School of Environmental and Life Sciences, College of Engineering Science and Environment, University of Newcastle, Callaghan, New South Wales, Australia

**Keywords:** stallion spermatozoa, artificial insemination, sperm isolation, stallion fertility, single layer centrifugation

## Abstract

**Graphical abstract:**

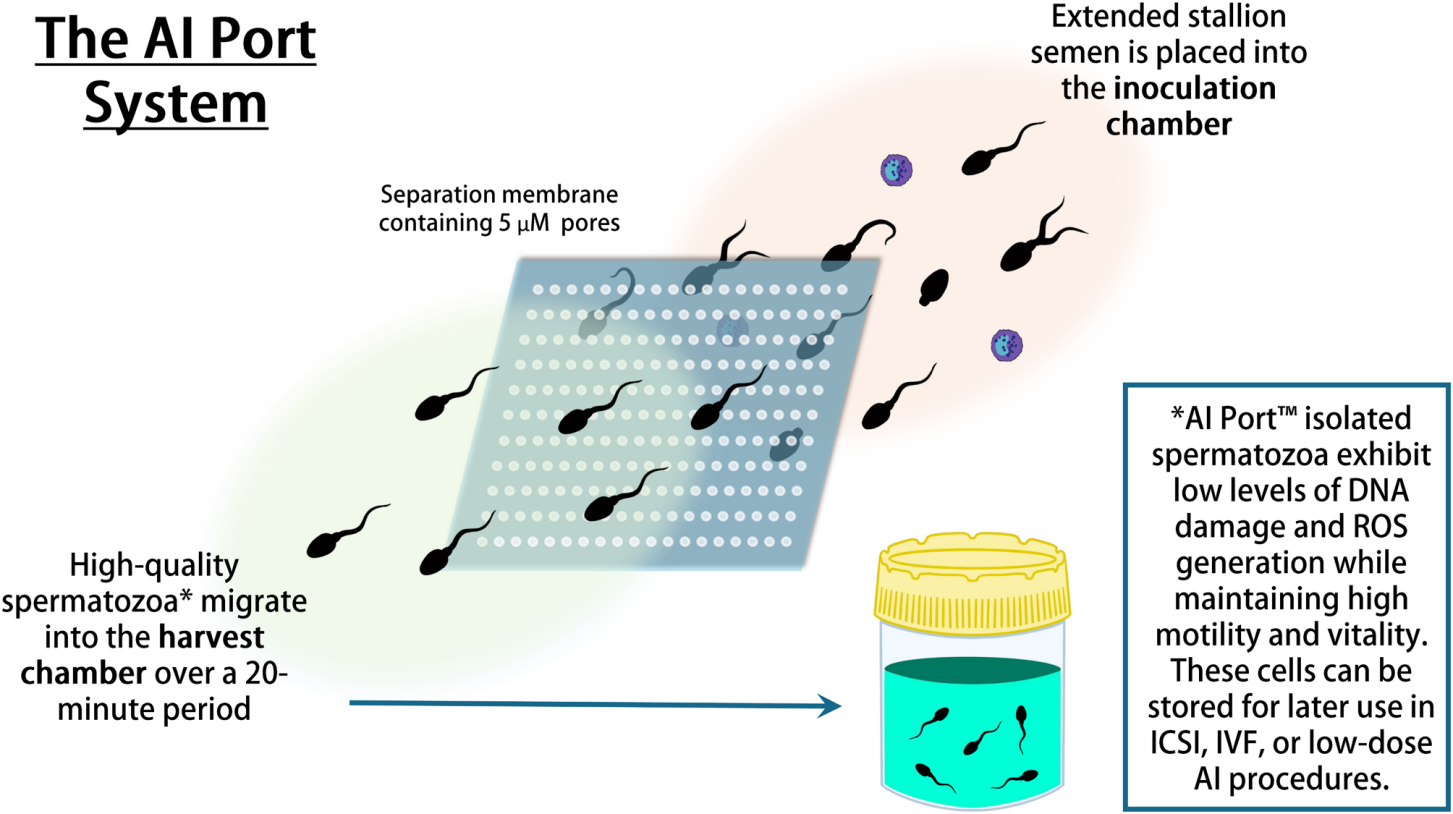

**Abstract:**

*In vitro* semen purification techniques have been developed that seek to mimic the *in vivo* selection process in order to generate the highest possible chance of oocyte fertilization following artificial insemination. Numerous methods have been developed to isolate functional spermatozoa for artificial insemination, yet only one method, single-layer centrifugation using commercial preparations like EquiPure, has been widely employed. In this study, we have introduced a novel approach for isolating spermatozoa and compared their quality to those isolated using EquiPure. The AI port system (Memphasys, Ltd. in Sydney, Australia) features a disposable cartridge with an inoculation chamber for depositing extended semen and a harvest chamber for extracting isolated spermatozoa. These chambers are separated by a 5 µm polyethylene terephthalate (PETE) membrane, allowing highly motile spermatozoa to migrate from the inoculation chamber to the harvest chamber over a 20-minute period. This migration effectively leaves behind seminal plasma and other cell types, such as leukocytes. Comparative analyses between spermatozoa isolated with the AI port and EquiPure demonstrated that, across all measured sperm parameters, including yield, vitality, motility, morphology, DNA fragmentation, and mitochondrial superoxide generation, the AI port-isolated cells exhibited comparable or superior performance, particularly in terms of DNA fragmentation. In summary, the AI port system demonstrates the potential to efficiently isolate high-quality spermatozoa, possibly offering a cost-effective and user-friendly alternative that may enhance the success rates of artificial insemination in breeding programs.

**Lay summary:**

This study aimed to create a new method for refining stallion semen to increase the likelihood of a successful pregnancy through artificial insemination. While there are existing techniques for isolating high-quality sperm, the most common involves a complicated process using a centrifuge, which spins the semen to separate it. This research introduces a new approach called the AI port system that uses a disposable cartridge with two separate chambers for putting in semen and getting out isolated sperm. A membrane between the chambers acts like a filter, letting highly motile sperm swim across, leaving behind unwanted substances like bacteria and blood cells. Compared to the centrifugation method, the AI port system effectively produces sperm with comparable or better quality in various aspects, including vitality, movement, shape, DNA integrity, and energy production. In summary, the AI port system is an easy-to-use alternative with the potential to improve the success of artificial insemination in horse breeding programs.

## Introduction

Equine artificial insemination (AI) offers numerous benefits, encompassing the elimination of the need to transport horses for breeding, mitigating the potential for sexually transmitted diseases, preventing breeding accidents, and enabling stallions to sire a notably larger number of mares compared to live cover. These advantages have stimulated the industry to actively pursue significant advancements in this field. Additionally, the current trend predominantly involves using chilled semen for AI, instead of fresh semen immediately after collection (Aurich 2005).

Removing a significant portion of the seminal plasma from the ejaculate could potentially yield positive outcomes, such as enhancing the stability of sperm membranes during chilled storage (Barrier-Battut *et al.* 2013). Moreover, there is a possibility that this action could lead to a reduction in chromatin damage (Lo *et al.* 2002), likely achieved by eliminating specific sources of reactive oxygen species (ROS) that pose a greater risk in the context of sperm preservation. Additionally, this practice may also have the potential to alleviate the inflammatory reaction in mares susceptible to post-breeding endometritis (Troedsson *et al.* 2001, Troedsson, 2006). In countries such as Germany, Austria (Pagl *et al.* 2006), and The Netherlands (Colenbrander *et al.* 2003), it is common practice to eliminate seminal plasma from the ejaculate through sperm washing (centrifugation), and subsequent resuspension in a skimmed milk-based semen extender. However, nations including the United Kingdom (Allen 2005), France (Batellier *et al.* 1998), and Italy (Rota *et al.* 2004) intentionally refrain from using this method due to concerns that the high g-forces involved in centrifugation may negatively impact sperm quality, potentially leading to harm to sperm chromatin. Utilizing colloid centrifugation has served as an alternative to sperm washing, offering the added advantage of selecting highly motile, morphologically sound, viable spermatozoa with intact chromatin integrity (Morrell *et al.* 2009). This technique also eliminates seminal plasma proteins that coat the surface of spermatozoa (Kruse *et al.* 2011), assisting cells in maintaining fertility even after 96 h of chilled storage (Lindahl *et al.* 2012). Although, it is important to note that cells still must undergo the centrifugation process.

Nonetheless, the widespread adoption of colloid centrifugation within the industry is limited, largely due to financial considerations. The processing of a single ejaculate can incur costs of several hundred dollars, further compounded by the requirement for specialized technicians to conduct the procedure. Hence, there arises a necessity to create a cost-efficient and user-friendly alternative for sperm processing, ultimately enhancing the success rates of artificial insemination using chilled semen in breeding programs.

Several devices have been developed to address the need for improved sperm separation technologies in the market. One such device is the Felix™ (Memphasys Ltd., Sydney, Australia), an electrophoretic separation device that has shown promising results in isolating both fresh and cryopreserved human spermatozoa. The Felix™ has been demonstrated to produce sperm samples with lower DNA damage and reduced levels of 4-HNE adduction when compared to traditional colloid centrifugation methods (Hungerford *et al.* 2023, Shapouri *et al.* 2023, Villeneuve *et al.* 2023). Despite its effectiveness with human spermatozoa, there is currently no literature on the use of the Felix™ with stallion spermatozoa.

Microfluidics has gained considerable traction for the application of sperm isolation. Notable devices include ZyMōt (ZyMōt Fertility Inc., Gaithersburg, MD, USA) and the VetCount™ Harvester (MotilityCount ApS, Valby, Denmark). The ZyMōt fertility chip has shown that it is able to isolate human spermatozoa with fewer double-strand DNA breaks and higher blastocyst rates when compared to swim-up and density gradient centrifugation methods (Pujol *et al.* 2022, Zaha *et al.* 2023). Likewise, it has also demonstrated the ability to isolate a more motile subpopulation of frozen/thawed stallion sperm from stallions of ‘good’ and ‘bad’ fertility (Vigolo *et al.* 2023). The VetCount™ Harvester currently has limited literature on the device’s success, with one study demonstrating that it is able to isolate more motile spermatozoa compared to single-layer centrifugation (Herbicht *et al.* 2023).

**Figure 1:**
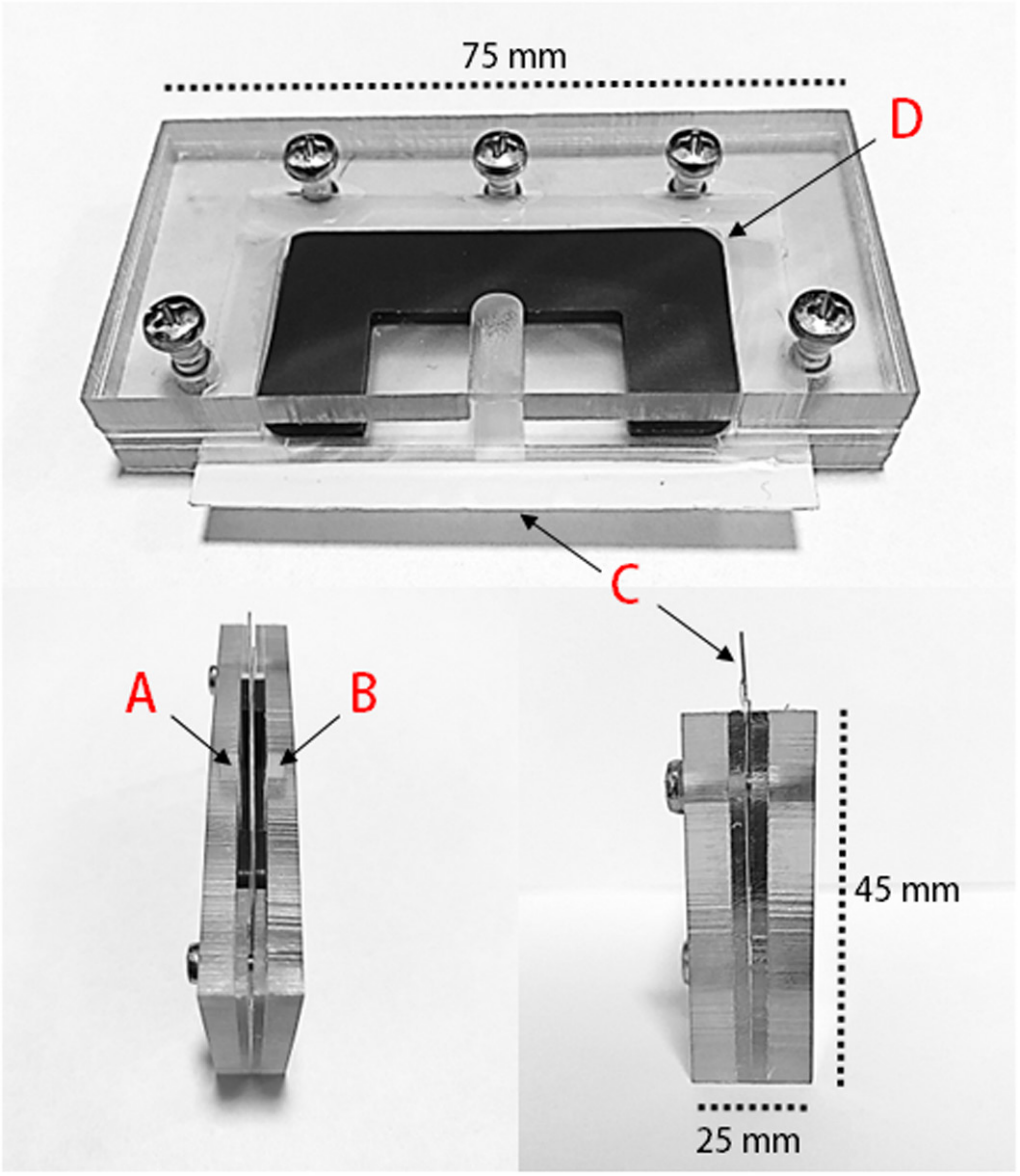
Stallion sperm isolation in the AI port system. (A) Inoculation chamber and (B) harvest chamber encased in an acrylic frame, separated by (C) a 5 µm polycarbonate filter and (D) a silicon seal. One mL of EquiPlus semen extender was deposited into the isolation chamber, followed by 1 mL of extended semen deposited into the inoculation chamber. The cartridge was kept at ambient temperature for 20 min, after which 1 mL was removed from the harvest chamber for analysis and subsequent storage.

Although the Felix™, ZyMōt, and the VetCount™ Harvester have shown an ability to isolate high-quality spermatozoa, their adoption in equine AI is limited by several factors. The associated costs of these devices and their limited semen processing capacity, while applicable for intracytoplasmic sperm injection (ICSI), make them less suitable for the demands of equine AI, which requires processing larger volumes of semen. The AI-port system (Memphasys Ltd., Sydney, Australia; Fig. 1) is designed to leverage the inherent motility of spermatozoa in order to isolate a subgroup of highly motile and hypothesized highly fertile cells. This prototype device consists of a disposable cartridge that houses an inoculation chamber for depositing extended semen and a harvest chamber for extracting isolated spermatozoa. These chambers are divided by a 5 µm PET membrane, allowing highly motile spermatozoa to migrate from the inoculation chamber to the harvest chamber within 20 minutes. During this process, seminal plasma and various other cell types, including leukocytes and bacteria, are left behind. Within the present study, we compared the effects of (i) no sperm isolation, (ii) single-layer centrifugation, and (iii) the AI port system on the quality of sperm before and after chilled storage. The overarching aim was to assess whether this approach to isolating spermatozoa by exploiting the intrinsic high motility of equine spermatozoa using the AI port system could generate sperm populations of equivalent or superior quality to those obtained through single-layer centrifugation.

## Materials and methods

### Materials

All chemicals used in this study were obtained from Sigma-Aldrich unless otherwise stated. A modified Biggers, Whitten, and Whittingham medium (BWW; Biggers *et al.,* 1971), containing 95 mM NaCl, 4.7 mM KCl, 1.7 mM CaCl_2_.2H_2_O, 1.2 mM KH_2_PO_4_, 1.2 mM MgSO_4_.7H_2_O, 25 mM NaHCO_3_, 5.6 mM D-Glucose, 275 µM C_3_H_3_NaO_3_, 3.7 µL/mL 60% NaC_3_H_5_O_3_ syrup, 50 U/mL penicillin, 50 µg/mL streptomycin, 20 mM HEPES, and 0.1% (w/v) polyvinyl alcohol, with an osmolarity of approximately 310 mOsm/kg and a pH of 7.4, was utilized throughout this study unless otherwise stated.

### Collection and preparation of stallion spermatozoa

Institutional Ethical Approval was secured for this project (A-2021-139). All experiments were conducted using multiple ejaculates from four normozoospermic miniature stallions, all of which were housed in facilities approved by the institution. Semen collection and extension procedures were performed following previously outlined protocols (Medica *et al.* 2021), and the collected semen was transported to the laboratory within a 2-h window from the time of collection.

For the purposes of this study, spermatozoa were categorized as follows: (i) unaltered ‘non-selected’ samples, extended in EquiPlus semen extender; (ii) isolated using EquiPure Gradients (Tek-Event Pty Ltd., Australia) as described by Medica *et al.* (2021), with the exception that only 1 mL of semen was overlaid on the EquiPure gradient to align the conditions with those of the AI port system; pelleted samples were then resuspended in 1 mL of BWW; and (iii) isolated using the AI port system (Memphasys Ltd, Sydney, Australia). The AI port system was run by placing 1 mL of semen into the inoculation chamber and 1 mL of EquiPlus semen extender into the harvest chamber (Fig. 1). After 20 min, 1 mL of the sample was removed from the harvest chamber and placed into a clean Eppendorf tube.

### Sperm chilling

After initial sperm assessments, samples were placed into a commercially available sperm-chilling Styrofoam box (Minitube, Australia) with two frozen ice bricks (~5°C). The ice bricks were changed every 12 h. Sperms were chilled for 48 h before secondary analysis (see 3.4).

### Sperm analysis

#### Yield

Immediately after isolation, sperm concentrations were determined using a NucleoCounter NC-100™ (ChemoMetec, Denmark). Samples were then diluted to approximately 10–15 × 10^6^ sperm/mL in Equiplus semen extender. Due to the size limitations of the current AI-port prototype, it currently cannot process large volumes of semen; therefore, the isolated concentration is less than that of Equipure. This concentration was chosen to ensure that both AI port and Equipure isolated cells were stored at the same concentration for direct comparison.

#### Motility

Computer-assisted sperm analysis (CASA; IVOS, Hamilton Thorne, Danvers, MA, USA) was utilized for the objective evaluation of sperm cell motility parameters. The following settings were applied: negative phase-contrast optics, a recording rate of 60 frames per second, a minimum cell size threshold of 5 µm², a maximum cell size threshold of 50 µm², a progressive average path velocity (VAP) threshold of 50 µm/s, a slow (static) cell VAP threshold of 20 µm/s, a slow (static) cell velocity (VSL) threshold of 0 µm/s, and a threshold straightness (STR) of 75%.

Sperm cells meeting the criteria of a VAP ≥50 μm/s and a STR ≥80% were categorized as progressive. For the analysis, immediately following isolation and once more after the chilling process, a volume of 3 µL of sperm from each sample was loaded into one chamber of a four-chambered slide with a depth measuring 20 µm (Leja; Gytech Pty Ltd, Australia). Throughout the analysis, the stage temperature was upheld at 37°C, and a minimum of 200 cells from five different fields were scrutinized for each sample.

#### Vitality

Following isolation and again after the chilling process, sperm viability was assessed through Eosin staining. A mixture of sperm solution and Eosin (0.5% Eosin Y in a 0.9% sodium chloride buffer) at a 1:1 ratio was applied to a microscope slide and covered with a coverslip. Samples were examined using a 40× objective, and the cells were classified as either alive (no staining) or dead (pink staining).

#### Morphology

Following isolation, spermatozoa from each treatment were promptly fixed with 2% paraformaldehyde for a duration of 10 min at a temperature of 5°C, washed with PBS, and stored in 0.1 M glycine in PBS for up to 1 week before further analysis. To assess cell morphology, an oil immersion 100× objective was used, and the presence or absence of cell defects was determined for 100 cells following cell fixation. The cells were classified into distinct categories, including: (i) normal, or displaying defects in the (ii) head, (iii) midpiece, (iv) tail, and as having cytoplasmic droplets at either the (v) proximal or (vi) distal end of the tail as previously described (Sieme 2009).

#### DNA fragmentation (HALO)

Following isolation, spermatozoa from each treatment were promptly diluted to 10 × 10^6^/mL before being snap-frozen in liquid nitrogen and stored at −80°C until analysis. Spermatozoa were thawed at room temperature and mixed with 1% low-melting agarose (final agarose concentration of 0.7%) at 37°C. A 50 µL aliquot of the agarose/sperm suspension was evenly spread onto a microscope slide that had been coated with 0.65% standard agarose. A coverslip was gently placed over the sample, which was then incubated at 5°C for 5 min. The slides were subsequently covered with 0.08 N HCl for 7 min at room temperature, with any residual solution being dabbed off onto a paper towel. The slides were then covered with Denaturation Solution 1 (0.4 M Tris, 1% SDS, 50 mM EDTA, 800 mM dithiothreitol, Milli-Q water, pH 7.5) for 10 min at room temperature, followed by Denaturation Solution 2 (0.4 M Tris, 1% SDS, 2 M NaCl, Milli-Q water, pH 7.5) for 5 min at room temperature, any residual solution being dabbed off onto a paper towel. The slides were next covered with 1× TBE buffer for 2 min at room temperature and then covered with 70% EtOH for 2 min at room temperature, any residual solution was dabbed off onto a paper towel. This step was repeated with 90% and 100% EtOH, before the slides were air-dried and finally stained with DAPI (1/2000) for 10 min at room temperature and rinsed with 1× PBS. For observation, the slides were mounted with 10 µL Mowiol and a coverslip before being imaged under a fluorescent microscope. A minimum of 100 cells were counted and classified for each treatment.

#### MitoSOX Red

Flow cytometry analyses were conducted using a FACSCanto II flow cytometer (Becton Dickinson, CA, USA) equipped with a 488-nm solid-state laser. Emission measurements were recorded using various filters, including a 530/30 nm bandpass (green/FITC), a 585/42 nm bandpass (red/PE), a >670 nm long-pass (far red/PerCp), and a 780/60 nm bandpass (far red/PECy7). To ensure accurate analysis, a gate was drawn and positioned around the sperm population while excluding debris, utilizing the forward scatter/side scatter dot plot. Each sample was analyzed with a minimum of 10,000 cells, and the data were processed using FACSDiva V8.01 software (Becton Dickinson). Spermatozoa were incubated at 37 ^o^C for 2 h before the MitoSOX Red (MSR) assay was run for both fresh and chilled samples to ensure enough intracellular superoxide had accumulated to be detected and compared between treatments.

To run the MSR assay, a 100 µL sperm suspension was initially washed with BWW via centrifugation (500 × ***g*** for 3 min), then subjected to a 15-min incubation at 37°C with 2 µM MSR stain (Molecular Probes) and 5 nM SYTOX™ Green (Molecular Probes) viability stain. The staining controls encompassed a positive dead control: 100 µL of spermatozoa underwent rapid freezing in liquid nitrogen, followed by a 15-min incubation at 37°C with 5 nM SYTOX™ Green viability stain only. Additionally, an MSR-positive control involved a 15-minute incubation of 100 µL of spermatozoa with 100 µM arachidonic acid (AA) and 2 µM MSR stain only. Subsequently, the spermatozoa were subjected to centrifugation at 500 × ***g*** for 3 min; the supernatant was then removed, and the pellets were resuspended in 300 µL BWW for subsequent analysis using FACSCanto II. It is worth noting that because all deceased cells register as positive for MSR (attributable to the presence of residual ethidium bromide within the MSR preparation), the data collected for statistical analysis exclusively pertain to the live-cell population.

### Statistical analysis

When deemed appropriate, one-way ANOVA was performed, followed by post hoc comparisons of group mean values against their respective controls using Dunnett’s test. Additionally, a MANOVA was employed to examine the association between the sperm isolation method and changes in sperm motility measured at two-time points, initially and after 48 h. This analysis allowed us to assess the multivariate effects of treatments across different time intervals. The selection of these parametric methods depended on the normality of data distribution, as assessed through the Anderson–Darling Goodness-of-Fit test. Concurrently, we verified the assumed homogeneity of variances using the Bartlett test. In cases where the data did not conform to a normal distribution, comparative non-parametric methods were used. Statistical significance was established when *P* ≤ 0.05. Where applicable, data were ‘blocked’ by individual stallions. The specific statistical tests and *n*-values utilized for each segment of this study are explicitly detailed in the figure legends. All data are presented in the form of means ± s.e.m.

## Results

**Figure 2:**
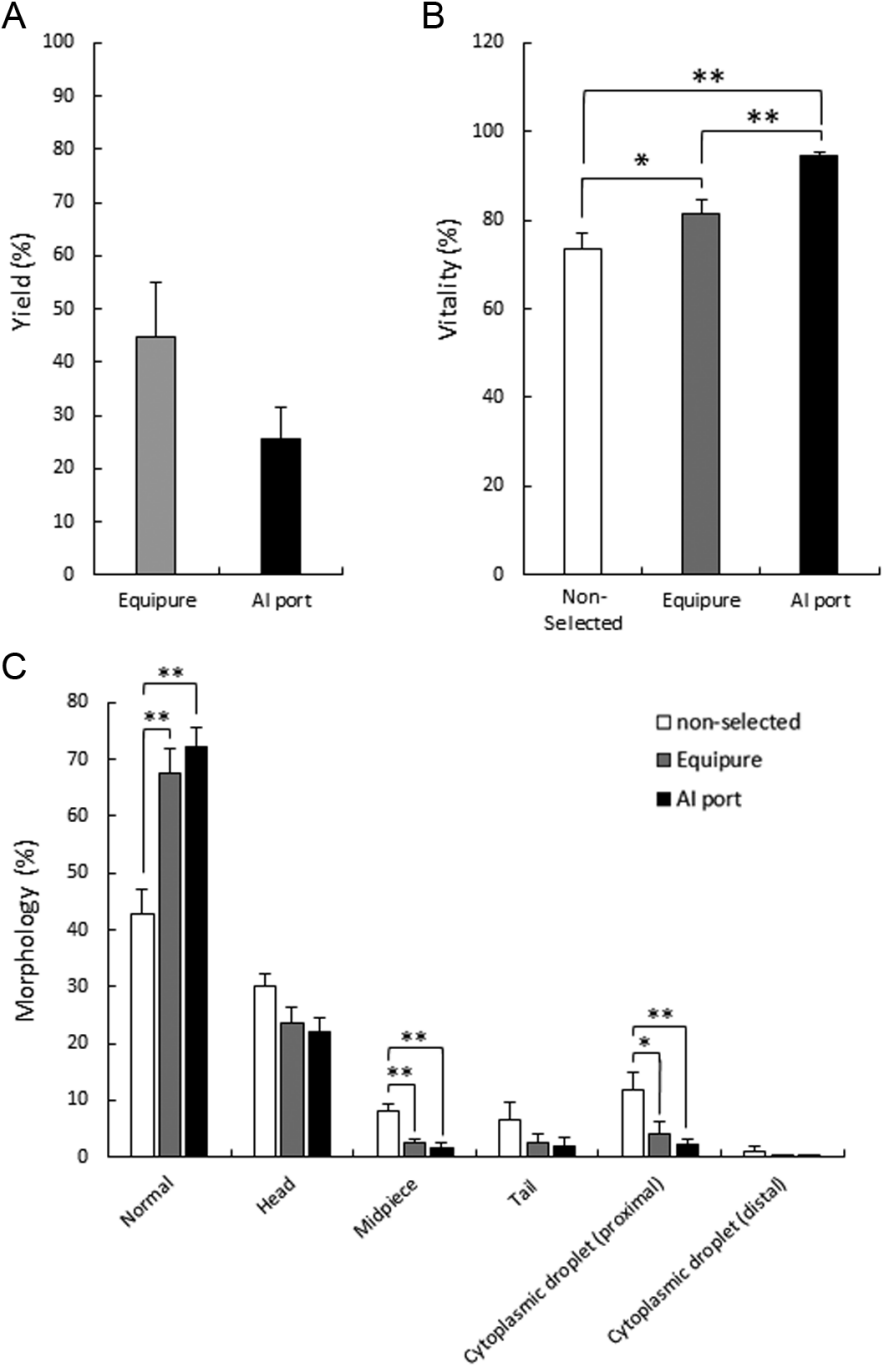
Parameters of stallion spermatozoa immediately after isolation. (A) Yield, expressed as the percentage isolated from the initial ejaculate. (B) Vitality, measured with Eosin, and (C) morphology of stallion spermatozoa measured after either no sperm isolation (non-selected), Equipure isolation, or AI port isolation (significant differences between each treatment were determined by ANOVA test; *n* = 10; *P < 0.05, **P < 0.01).

Following the collection of semen and its subsequent extension, stallion sperm cells were either classified as ‘non-selected’ (extended in Equiplus semen extender only) or separated using either Equipure gradients or the AI port system. The resulting yield from each method was recorded as the percentage of sperm cells isolated from the parent non-selected sample (Fig. 2A). When comparing both methods of sperm isolation, there was no significant difference in the yield of spermatozoa from either Equipure or the AI port system (44.9 ± 10.1% vs 25.5 ± 5.9% respectively).

Immediately after sperm isolation, vitality was assessed via eosin staining (Fig. 2B). Comparisons were made with the non-selected sample (73.6 ± 3.4%), and both Equipure (81.5 ± 3.1%; *P* ≤ 0.05) and the AI port system (94.4 ± 1.1%; *P* ≤ 0.01) were found to significantly enhance the vitality of the sample. It is noteworthy that the vitality of the AI port system was also significantly greater than that of the Equipure sample (*P* ≤ 0.01).

Similarly, following sperm isolation, cell morphology was assessed (Fig. 2C). This evaluation encompassed two key aspects: the percentage of cells categorized as normal and the percentage of cells exhibiting abnormalities, which were further categorized into defects located in the head, midpiece, and tail regions, as well as the presence of proximal or distal cytoplasmic droplets. Compared to the non-selected sample, both Equipure (*P* ≤ 0.01) and AI port-(*P* ≤ 0.01) isolated cells exhibited a significant increase in the proportion of morphologically normal cells (42.7 ± 4.3% vs 67.4 ±4.5% and 72.3 ± 3.4%, respectively). Furthermore, midpiece defects were significantly reduced in both Equipure (*P* ≤ 0.01) and AI port-(*P* ≤ 0.01) isolated cells (8 ± 1.3% vs 2.4 ± 0.7% and 1.5 ± 1.6%, respectively). Likewise, a significant decrease in proximal cytoplasmic droplets was observed in cells isolated through both Equipure (*P* ≤ 0.05) and the AI port system (*P* ≤ 0.01) when compared to the non-selected samples (11.8 ± 13.1% vs 4.1 ± 2.2% and 2.3 ± 0.7%, respectively). Across all treatments, there was no significant difference in the occurrence of cells with head or tail defects, nor in the presence of cytoplasmic droplets (distal) among the cells.

**Figure 3:**
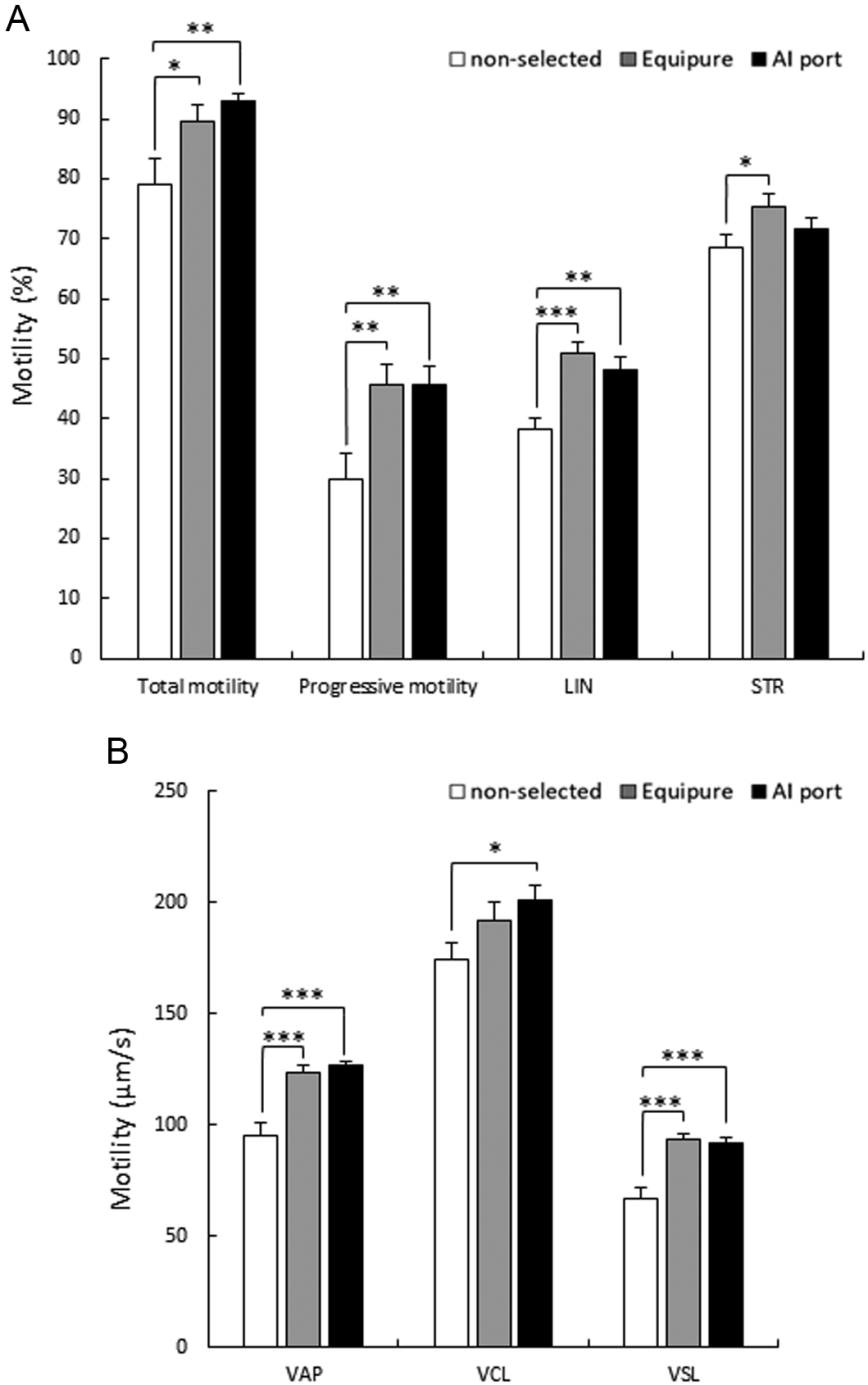
Motility parameters of stallion spermatozoa immediately after isolation. (A) Total motility, progressive motility, linearity (LIN), and straightness (STR); and (B) average path velocity (VAP), curvilinear velocity (VCL), and straight-line velocity (VSL) of stallion spermatozoa measured after either no sperm isolation (non-selected), Equipure isolation, or AI port isolation (significant differences between each treatment were determined by non-parametric Wilcoxon test; *n* = 10; *P < 0.05, **P < 0.01, ***P < 0.001).

Sperm motility (Fig. 3A), measured via CASA immediately after isolation, indicated that compared to the non-selected sample, both Equipure (*P* ≤ 0.05) and AI port-(*P* ≤ 0.01) isolated cells displayed significantly higher total motility (78.9 ± 4.6% vs 89.6 ± 2.9% and 93.2 ± 1.3% respectively). This trend was similarly observed for progressive motility (30.0 ± 4.0% vs 45.6 ± 3.4% and 45.7 ± 3.2%; *P* ≤ 0.01 and *P* ≤ 0.01, respectively) and linearity (38.3 ± 1.8% vs 50.8 ± 2.05% and 48.0 ± 2.1%; *P* ≤ 0.001 and *P* ≤ 0.01, respectively). Regarding straightness, compared to the non-selected sample, Equipure-isolated cells displayed a significantly straighter motility pattern (68.6 ± 2.1% vs 75.4 ± 2.1; respectively; *P* ≤ 0.05), whereas the AI port-isolated cells showed no significant difference (71.5 ± 1.8%).

Velocity parameters of sperm (Fig. 3B) were measured by CASA immediately after isolation. When compared to the non-selected sample, Equipure (*P* ≤ 0.001) and AI port-(*P* ≤ 0.001) isolated cells displayed a significant increase in both average path velocity (VAP; 94.8 ± 6.0 µm/s vs 123.2 ± 3.7 µm/s and 126.5 ± 2.1 µm/s, respectively) and straight-line velocity (VSL; 66.8 ± 4.7 µm/s vs 93.1 ± 2.9 µm/s and 91.7 ± 2.8 µm/s). Compared to the non-selected sample, the AI port-isolated sample showed a significant increase in curvilinear velocity (VCL; 173.9 ± 7.8 µm/s vs 201.0 ± 6.7 µm/s; *P* ≤ 0.05). In contrast, the Equipure-isolated sample did not exhibit a significant improvement compared to the non-selected sample (191.9 ± 8.1 µm/s).

**Figure 4:**
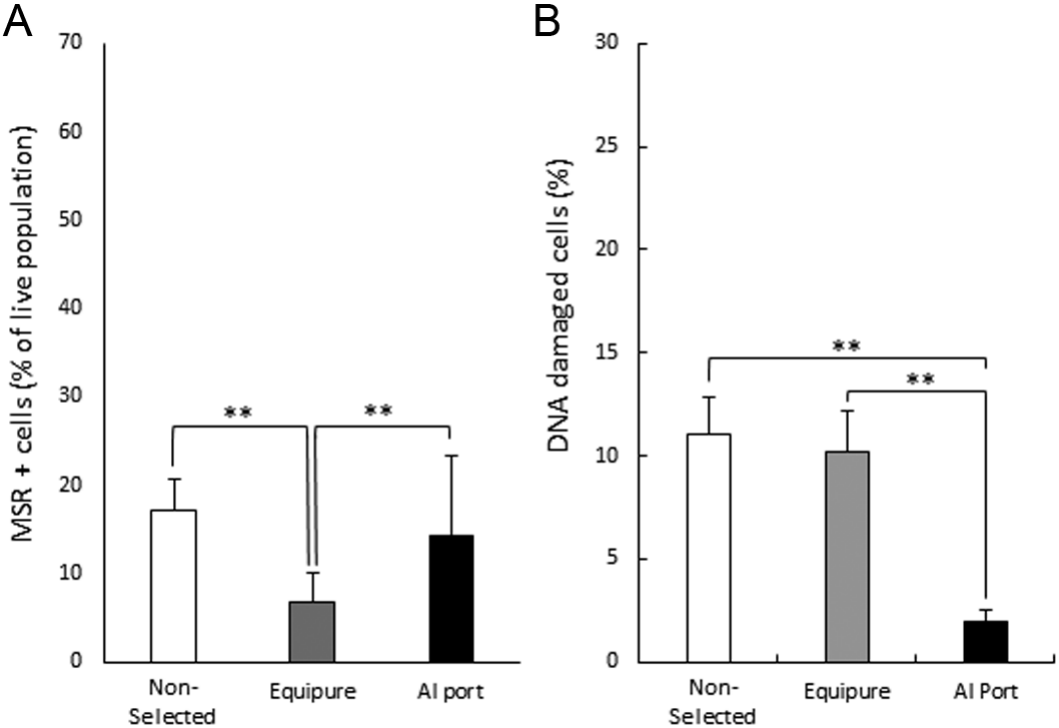
Intracellular superoxide generation and DNA fragmentation of stallion spermatozoa. (A) Intracellular superoxide generation was measured with MitoSOX Red (MSR), and (B) DNA fragmentation was measured with the HALO DNA dispersion test of stallion spermatozoa measured after either no sperm isolation (non-selected), Equipure isolation or AI port isolation (significant differences between each treatment were determined by ANOVA test; *n* = 10; **P < 0.01).

Intracellular superoxide production was evaluated using flow cytometry and the MitoSOX Red assay. Cells were isolated and placed in a 37 °C incubator for 2 h before conducting the MSR assay (Fig. 4A). The samples isolated using Equipure (6.7 ± 3.4%; *P* ≤ 0.01) demonstrated a significant decrease in intracellular superoxide production when compared with both the non-selected samples (17.2 ± 3.5%) and the AI port-isolated samples (14.3 ± 8.9%). Notably, there was no significant difference between the non-selected samples and those isolated with the AI port system.

DNA fragmentation was assessed with the HALO dispersion test (Fig. 4B). AI port-isolated cells exhibited a substantial decrease in DNA fragmentation when compared to both the non-selected cells and the cells isolated using Equipure (2 ± 0.5% vs 11 ± 1.9% vs 10.2 ± 2.0%, respectively; *P* ≤ 0.01). Notably, there was no significant difference between the non-selected samples and those isolated with Equipure.

**Figure 5:**
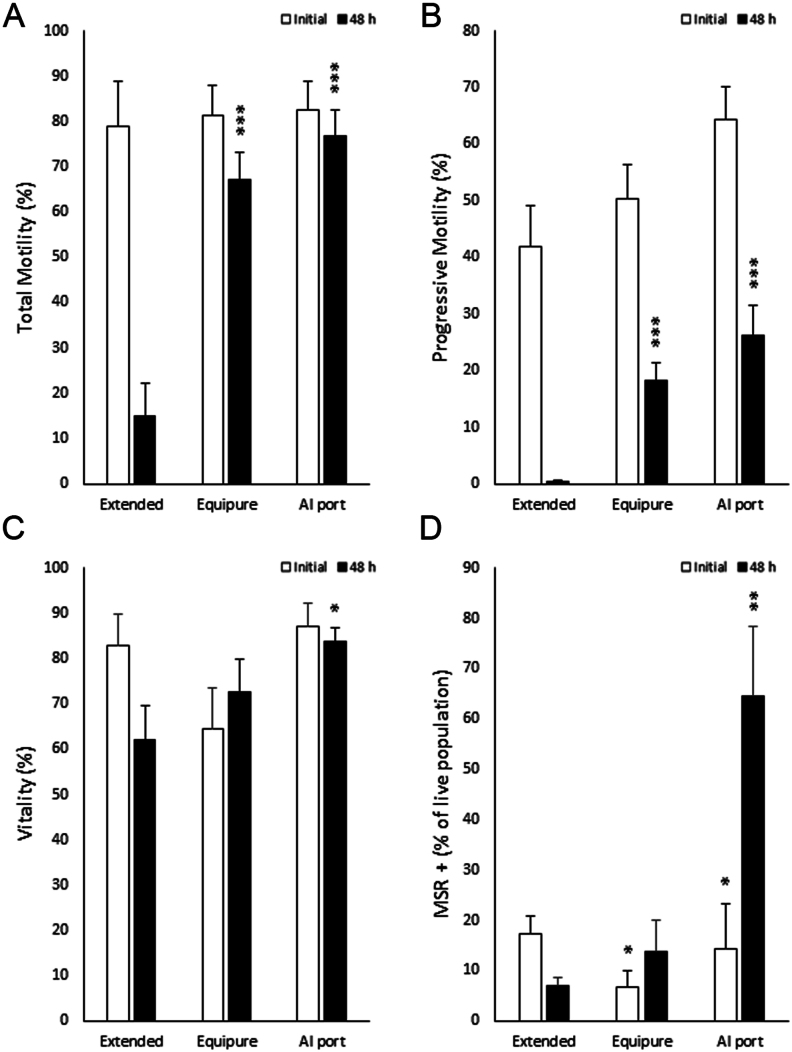
Quality assessment of stallion spermatozoa after 48 h chilled storage. (A) Total motility, (B) progressive motility, (C) vitality, and (D) intracellular superoxide production measured with MitoSox Red (MSR) of stallion spermatozoa after either no sperm isolation (non-selected), Equipure isolation, or AI port isolation and 48 h chilled storage at 5°C (significant differences between each treatment were determined by ANOVA test; *n* = 9; *P < 0.05, **P < 0.01, ***P < 0.001).

As the overarching aim for spermatozoa isolated through the AI port device is to preserve them through chilled storage for future artificial insemination, spermatozoa were isolated using either Equipure gradients or the AI port system and exposed to a 48-h period of chilling at 5 °C. Following the chilling process, the quality of the cells was re-evaluated to assess the ability of the spermatozoa to maintain their quality during these storage conditions. The MANOVA analysis provided critical insights into the effectiveness of the isolation methods over time. Significant differences in total and progressive motility between the isolation methods indicated that treatment effects were not merely dependent on the type of isolation but also varied significantly over time. Specifically, cells isolated using the AI port system exhibited higher total motility compared to Equipure (76.8 ± 7.1% vs 67.2 ± 6.9%; respectively; *P* ≤ 0.01) (Fig. 5A) and higher progressive motility compared to Equipure (26.1 ± 5.4% vs 18.2 ± 3.3; respectively; *P* ≤ 0.01) (Fig. 5B), demonstrating the superior efficacy of the AI port system in preserving motility during chilling.

Furthermore, the vitality assessments post-chilling showed an increase only in the AI port-isolated cells (83.1 ± 3.0%; *P* ≤ 0.05). In contrast, cells isolated with Equipure did not show a substantial improvement in vitality (72.7 ± 7.1%) when compared to the non-selected sample (62.1 ± 7.6%), suggesting that the AI port method significantly enhances the ability of spermatozoa to maintain their vitality under prolonged storage conditions (Fig. 5C). This finding was supported by the interaction effects in the MANOVA, highlighting a treatment-specific response over time (*P* ≤ 0.01).

Additionally, the analysis of ROS generation revealed a significant increase in mitochondrial ROS in cells isolated via the AI port compared to non-selected and Equipure-isolated cells (64.5 ± 13.8% vs 6.8 ± 1.8% vs 13.8 ± 6.1%; respectively; *P* ≤ 0.01). This significant finding underscores the nuanced impact of the isolation technique on cellular oxidative stress, further validated by the MANOVA, which pointed to significant interaction effects of time and treatment on the generation of mitochondrial ROS (*P* ≤ 0.01) (Fig. 5D).

## Discussion

In this study, we compared the impact of three different sperm processing treatments: (i) no sperm isolation, (ii) single-layer centrifugation (Equipure); and (iii) the AI port prototype device on sperm quality parameters, including yield, motility, vitality, morphology, DNA fragmentation, and intracellular superoxide generation, both pre- and post-chilled storage. The findings indicate that the AI port system can isolate cells of comparable or superior quality when compared to single-layer centrifugation (Equipure), and this quality can also be preserved even after 48 h of chilled storage.

Whilst there was no statistical difference in the yields achieved by single-layer centrifugation and the AI port system, a substantially lower percentage was recorded with the latter. It is acknowledged that the quantity of motile spermatozoa inseminated into the mare directly correlates with the likelihood of achieving a successful pregnancy. Indeed, it has been recorded that a concentration of >300 × 10^6^ progressively motile spermatozoa is generally required for traditional inseminations using fresh semen (Gahne *et al.* 1998). Consequently, the relatively lower yield observed in the current AI port setup may discourage potential users. Presently, the AI port incorporates a PETE membrane with 5 µm pores that are not uniformly distributed across the membrane, leading to a reduction in yield. This is due to the spermatozoa having to come into direct contact with a pore to pass through into the harvest chamber. To enhance yield, it might be possible to replace the PETE membranes with mesh-style membranes that feature more uniformly spaced pores, reducing the ‘dead’ space between them. Notwithstanding such potential modifications, it should be acknowledged that maximizing yield may involve a trade-off with sperm quality. Under present circumstances, as the AI port-isolated spermatozoa demonstrate comparable or superior quality, particularly concerning vitality and DNA fragmentation, attributes that have previously been linked to pregnancy outcome (Morrell *et al.* 2008, Atroshchenko *et al.* 2019), early embryo death, and late term abortions (Kumar *et al.* 2012, Peña *et al.* 2017). Therefore, the principle ‘quality over quantity’ may well apply when considering the relative value of these sperm preparation methods.

Typically, the initial step in evaluating semen quality involves the assessment of sperm motility. Although motility does not directly correlate with fertility (Jasko *et al.* 1992, Wilhelm *et al.* 1996), an immotile cell cannot fertilize an oocyte. The AI port exploits the high motility of stallion spermatozoa in order to isolate only the most motile cells. In all motility assessments (total, progressive, LIN, STR, VAP, VCL, and VSL), cells isolated with the AI port performed either on par or significantly better than Equipure-isolated cells. Importantly, this motility was maintained even after chilled storage, whereas the non-selected samples incubated under identical conditions experienced an almost complete loss of motility. It is important to note that the non-selected samples still contained high concentrations of seminal plasma, which is known to be highly toxic to spermatozoa during storage (Todd *et al.* 2001, Morrell *et al.* 2010, Kruse *et al.* 2011). Yet, there is supporting evidence suggesting that small quantities of seminal plasma can be advantageous during storage, as it contains protective decapacitation factors (Leahy & Gadella 2011) and enhances immune interactions with the female reproductive tract (Robertson 2007). While single-layer centrifugation is capable of completely eliminating the entire seminal plasma component as part of its inherent design (Kruse *et al.* 2011), in the case of the AI port, some seminal plasma may cross the membrane via diffusion, which may potentially have advantageous effects.

Superficially, the significantly higher levels of superoxide generated in the AI port samples after 48 h of chilled storage may appear to reflect negatively on this sperm isolation system. Paradoxically, it is now widely recognized that stallion spermatozoa heavily depend on oxidative phosphorylation (OXPHOS; Morrell *et al.* 2008, Ortega Ferrusola *et al.* 2010, Gibb *et al.* 2014), unlike other species such as humans, who primarily rely on glycolysis (Storey 2008). The advantage of OXPHOS lies in the comparatively high production of ATP compared to glycolysis, allowing stallion spermatozoa to sustain their motility, maturation, and subsequent capacitation. This reliance on the mitochondria leads to the accumulation of ROS (Halliwell & Gutteridge 2015). Stallion sperm atozoa have evolved to efficiently manage these heightened ROS levels via the evolution of a series of protective measures, including a highly effective aldehyde dehydrogenase system (Gibb *et al.* 2016). Therefore, it has been concluded that unless other vital sperm parameters such as motility and vitality are significantly affected, high mitochondrial superoxide levels indicate that the cell is exceptionally metabolically active and, as a result, highly fertile (Gibb *et al.* 2014).

## Conclusions

In this study, we introduced a device aimed at improving the preparation of spermatozoa for chilled storage and subsequent artificial insemination. The AI port system offers a significantly more cost-effective and user-friendly alternative to the industry’s current gold standard, single-layer centrifugation using commercially available preparations like Equipure. Our results demonstrate that spermatozoa processed with the AI port system exhibit equivalent or superior quality in key metrics such as viability, motility, ROS generation, and DNA fragmentation. Although our study did not directly evaluate fertility outcomes through artificial insemination, the quality metrics assessed are well-established indicators of fertility potential. Consequently, the AI port system holds significant promise for enhancing the efficiency of equine breeding programs. However, to fully validate the efficacy and practical application of this method, further field trials focusing on fertility outcomes are warranted. These trials will be essential for providing insights into the reproducibility and scalability of the AI port system, ultimately determining its viability as a potential tool for improving stallion fertility rates within the equine industry.

## Declaration of interest

The financial support for the Ph.D. student who conducted these studies, AJ Medica, was granted through a scholarship awarded by Memphasys Ltd, a biotechnology company specializing in cell separation technologies.

## Funding

This work was supported by Memphasys Ltd., Sydney, Australia.

## Author contribution statement

AM assisted with stallion semen collections, conducted the experiments, performed statistical analysis, and wrote the manuscript. ZG assisted with stallion semen collections. RJA conceived, funded the study, reviewed, and edited the manuscript.

## References

[bib1] Allen WR 2005 The development and application of the modern reproductive technologies to horse breeding. Reproduction in Domestic Animals 40 310–329. (10.1111/j.1439-0531.2005.00602.x) 16008761

[bib2] Atroshchenko MM Arkhangelskaya E Isaev DA Stavitsky SB Zaitsev AM Kalaschnikov VV Leonov S & Osipov AN 2019 Reproductive characteristics of thawed stallion sperm. Animals 9 1099. (10.3390/ani9121099) 31818047 PMC6940853

[bib3] Aurich C 2005 Factors affecting the plasma membrane function of cooled-stored stallion spermatozoa. Animal Reproduction Science 89 65–75. (10.1016/j.anireprosci.2005.06.025) 16081230

[bib4] Barrier-Battut I Bonnet C Giraudo A Dubois C Caillaud M & Vidament M 2013 Removal of seminal plasma enhances membrane stability on fresh and cooled stallion spermatozoa. Reproduction in Domestic Animals 48 64–71. (10.1111/j.1439-0531.2012.02026.x) 22524671

[bib5] Batellier F Duchamp G Vidament M Arnaud G Palmer E & Magistrini M 1998 Delayed insemination is successful with a new extender for storing fresh equine semen at 15°C under aerobic conditions. Theriogenology 50 229–236. (10.1016/s0093-691x(98)00130-7) 10734490

[bib6] Biggers J Whitten W Whittingham D & Daniel J 1971 Methods in Mammalian Embryology, pp. 86–116. San Francisco, CA: Freeman.

[bib7] Colenbrander B Gadella BM & Stout TA 2003 The predictive value of semen analysis in the evaluation of stallion fertility. Reproduction in Domestic Animals 38 305–311. (10.1046/j.1439-0531.2003.00451.x) 12887569

[bib8] Gahne S Gåneheim A & Malmgren L 1998 Effect of insemination dose on pregnancy rate in mares. Theriogenology 49 1071–1074. (10.1016/s0093-691x(98)00055-7) 10732114

[bib9] Gibb Z Lambourne SR & Aitken RJ 2014 The paradoxical relationship between stallion fertility and oxidative Stress1. Biology of Reproduction 91. (10.1095/biolreprod.114.118539) 25078685

[bib10] Gibb Z Lambourne SR Curry BJ Hall SE & Aitken RJ 2016 Aldehyde dehydrogenase plays a pivotal role in the maintenance of stallion sperm motility. Biology of Reproduction 94 133. (10.1095/biolreprod.116.140509) 27103446

[bib11] Halliwell B & Gutteridge JMC 2015 Free Radicals in Biology and Medicine. Oxford: Oxford University Press. (10.1093/acprof:oso/9780198717478.001.0001)

[bib12] Herbicht R Neufeld G Klein C & Henning H 2023 Evaluation of a novel microfluidic chip-like device for purifying bovine frozen-thawed semen for in vitro fertilization. Theriogenology 209 50–59. (10.1016/j.theriogenology.2023.06.019) 37356279

[bib13] Hungerford AJ Bakos HW & Aitken RJ 2023 Analysis of sperm separation protocols for isolating cryopreserved human spermatozoa. Reproduction and Fertility 4. (10.1530/RAF-22-0133) PMC10160538 37000632

[bib14] Jasko DJ Little TV Lein DH & Foote RH 1992 Comparison of spermatozoal movement and semen characteristics with fertility in stallions: 64 cases (1987–1988). Journal of the American Veterinary Medical Association 200 979–985. (10.2460/javma.1992.200.07.979) 1577655

[bib15] Kruse R Dutta PC & Morrell JM 2011 Colloid centrifugation removes seminal plasma and cholesterol from boar spermatozoa. Reproduction, Fertility, and Development 23 858–865. (10.1071/RD10260) 21871205

[bib16] Kumar K Deka D Singh A Mitra DK Vanitha BR & Dada R 2012 Predictive value of DNA integrity analysis in idiopathic recurrent pregnancy loss following spontaneous conception. Journal of Assisted Reproduction and Genetics 29 861–867. (10.1007/s10815-012-9801-3) 22692280 PMC3463671

[bib17] Leahy T & Gadella BM 2011 Sperm surface changes and physiological consequences induced by sperm handling and storage. Reproduction 142 759–778. (10.1530/REP-11-0310) 21964828

[bib18] Lindahl J Dalin A-M Stuhtmann G & Morrell JM 2012 Stallion spermatozoa selected by single layer centrifugation are capable of fertilization after storage for up to 96 h at 6°C prior to artificial insemination. Acta Veterinaria Scandinavica 54 40. (10.1186/1751-0147-54-40) 22788670 PMC3537575

[bib19] Lo CC Thompson JA Lowry VK & Varner DD 2002 Effect of storage time and temperature on stallion sperm DNA and fertility. Theriogenology 57 1135–1142. (10.1016/s0093-691x(01)00689-6) 12041906

[bib20] Medica AJ Aitken RJ Nicolson GL Sheridan AR Swegen A De Iuliis GN & Gibb Z 2021 Glycerophospholipids protect stallion spermatozoa from oxidative damage in vitro. Reproduction and Fertility 2 199–209. (10.1530/RAF-21-0028) 35118390 PMC8801026

[bib21] Morrell JM Johannisson A Dalin AM Hammar L Sandebert T & Rodriguez-Martinez H 2008 Sperm morphology and chromatin integrity in Swedish Warmblood stallions and their relationship to pregnancy rates. Acta Veterinaria Scandinavica 50 2. (10.1186/1751-0147-50-2) 18179691 PMC2246141

[bib22] Morrell JM Johannisson A Dalin AM & Rodriguez-Martinez H 2009 Morphology and chromatin integrity of stallion spermatozoa prepared by density gradient and single layer centrifugation through silica colloids. Reproduction in Domestic Animals 44 512–517. (10.1111/j.1439-0531.2008.01265.x) 18992087

[bib23] Morrell JM Rodriguez-Martinez H & Johannisson A 2010 Single layer centrifugation of stallion spermatozoa improves sperm quality compared with sperm washing. Reproductive Biomedicine Online 21 429–436. (10.1016/j.rbmo.2010.03.027) 20674501

[bib24] Ortega Ferrusola C Gonzalez Fernandez L Salazar Sandoval C Macias Garcis B Rodriguez-Martinez H Tapia JA & Peña FJ 2010 Inhibition of the mitochondrial permeability transition pore reduces “apoptosis like” changes during cryopreservation of stallion spermatozoa. Theriogenology 74 458–465. (10.1016/j.theriogenology.2010.02.029) 20451990

[bib25] Pagl R Aurich JE Muller-Schlosser F Kankofer M & Aurich C 2006 Comparison of an extender containing defined milk protein fractions with a skim milk-based extender for storage of equine semen at 5 degrees C. Theriogenology 66 1115–1122. (10.1016/j.theriogenology.2006.03.006) 16620943

[bib26] Peña ST Gummow B Parker AJ & Paris DBBP 2017 Revisiting summer infertility in the pig: could heat stress-induced sperm DNA damage negatively affect early embryo development? Animal Production Science 57 1975–1983. (10.1071/AN16079)

[bib27] Pujol A García-Peiró A Ribas-Maynou J Lafuente R Mataró D & Vassena R 2022 A microfluidic sperm-sorting device reduces the proportion of sperm with double-stranded DNA fragmentation. Zygote 30 200–205. (10.1017/S0967199421000484) 34313213

[bib28] Robertson SA 2007 Seminal fluid signaling in the female reproductive tract: lessons from rodents and pigs. Journal of Animal Science 85(Supplement) E36–E44. (10.2527/jas.2006-578) 17085725

[bib29] Rota A Furzi C Panzani D & Camillo F 2004 Studies on motility and fertility of cooled stallion spermatozoa. Reproduction in Domestic Animals 39 103–109. (10.1111/j.1439-0531.2004.00488.x) 15065992

[bib30] Shapouri F Mahendran T Govindarajan M Xie P Kocur O Palermo GD Bakos HW Ahlström A Caisander G Xu B, *et al.* 2023 A comparison between the Felix™ electrophoretic system of sperm isolation and conventional density gradient centrifugation: a multicentre analysis. Journal of Assisted Reproduction and Genetics 40 83–95. (10.1007/s10815-022-02680-0) 36515800 PMC9840737

[bib31] Sieme H 2009 Chapter 6 - Semen evaluation. In Equine Breeding Management and Artificial Insemination, 2nd ed. Ed Samper JC. Saint Louis: W.B. Saunders.

[bib32] Storey BT 2008 Mammalian sperm metabolism: oxygen and sugar, friend and foe. International Journal of Developmental Biology 52 427–437. (10.1387/ijdb.072522bs) 18649255

[bib33] Todd P Aarns MJ Chenoweth P & Schultz B 2001 Influence of seminal plasma and processing on cold-stored stallion spermatozoa. Animal Reproduction Science 68 335–336.

[bib34] Troedsson MHT 2006 Breeding-induced endometritis in mares. Veterinary Clinics of North America: Equine Practice 22 705–712. (10.1016/j.cveq.2006.07.003) 17129798

[bib35] Troedsson MHT Loset K Alghamdi AM Dahms B & Crabo BG 2001 Interaction between equine semen and the endometrium: the inflammatory response to semen. Animal Reproduction Science 68 273–278. (10.1016/s0378-4320(01)00164-6) 11744271

[bib36] Vigolo V Gautier C Falomo ME & Aurich C 2023 Selection of frozen-thawed stallion semen by microfluidic technology. Reproduction in Domestic Animals 58 443–449. (10.1111/rda.14305) 36510754

[bib37] Villeneuve P Saez F Hug E Chorfa A Guiton R Schubert B Force A & Drevet JR 2023 Spermatozoa isolation with Felix™ outperforms conventional density gradient centrifugation preparation in selecting cells with low DNA damage. Andrology 11 1593–1604. (10.1111/andr.13384) 36629014

[bib38] Wilhelm KM Grahem JK & Squires EL 1996 Comparison of the fertility of cryopreserved stallion spermatozoa with sperm motion analyses, flow cytometric evaluation, and zona-free hamster oocyte penetration. Theriogenology 46 559–578. (10.1016/0093-691X(96)00209-9) 16727923

[bib39] Zaha I Naghi P Stefan L Bunescu C Radu M Muresan ME Sandor M Sachelarie L & Huniadi A 2023 Comparative study of sperm selection techniques for pregnancy rates in an unselected IVF-ICSI population. Journal of Personalized Medicine 13 619. (10.3390/jpm13040619) 37109005 PMC10145657

